# Draft Genome Sequences of Fungi Isolated from the International Space Station during the Microbial Tracking-2 Experiment

**DOI:** 10.1128/MRA.00751-21

**Published:** 2021-09-16

**Authors:** Anna C. Simpson, Camilla Urbaniak, John R. Bateh, Nitin K. Singh, Jason M. Wood, Marilyne Debieu, Niamh B. O’Hara, Jos Houbraken, Christopher E. Mason, Kasthuri Venkateswaran

**Affiliations:** a Jet Propulsion Laboratory, California Institute of Technology, Pasadena, California, USA; b Biotia, New York, New York, USA; c Department of Cell Biology, College of Medicine, SUNY Downstate Health Sciences University, Brooklyn, New York, USA; d Westerdijk Fungal Biodiversity Institute, Utrecht, the Netherlands; e Department of Physiology and Biophysics, Weill Cornell Medicine, New York, New York, USA; f WorldQuant Initiative for Quantitative Prediction, Weill Cornell Medicine, New York, New York, USA; University of Maryland School of Medicine

## Abstract

As part of the Microbial Tracking-2 study, 94 fungal strains were isolated from surfaces on the International Space Station, and whole-genome sequences were assembled. Characterization of these draft genomes will allow evaluation of microgravity adaption, risks to human health and spacecraft functioning, and biotechnological applications of fungi.

## ANNOUNCEMENT

Fungi are potential sources of nutrients and bioactive compounds during long-term spaceflight but also could affect astronaut health through both opportunistic infections and system biofouling (1, 2). As part of a study characterizing fungal responses to the space environment, we report the draft genomes of 94 fungal strains that were isolated from the International Space Station (ISS), representing 10 ascomycetous and 1 basidiomycetous species.

Aspergillus species are environmental fungi and opportunistic pathogens (3). Aspergillus unguis is a member of the ISS microbiome (4) and produces industrially important compounds (5). Aureobasidium pullulans is a black fungus that was previously isolated from the ISS water filtration system (6) and Mars mission spacecraft-associated surfaces (7).

*Cladosporium* species are dominant fungal contaminants in indoor air (8, 9). Cladosporium sphaerospermum and Cladosporium cladosporioides were detected multiple time on the ISS, and their properties in microgravity were studied (10, 11).

Fusarium veterinarium is a recently described species within the Fusarium oxysporum complex, the species of which are ubiquitous in soil, are known human/plant pathogens (12), and were isolated both from surfaces and from infected Zinnia hybrida plants aboard the ISS (13, 14). Fusarium annulatum, which has been isolated from plant and human tissues on Earth, has not been reported previously in space (15).

*Penicillium* species produce important bioactive compounds and can contaminate food and cause secondary infections (16). Previously detected on the ISS or Mir (2, 11) are Penicillium citrinum, a common soil and indoor species (17), Penicillium rubens, from which penicillin was isolated (18), and Penicillium corylophilum, which is commonly found in damp buildings (19). Penicillium palitans, which has been reported in cheese (20) and also in a wide range of habitats, including Antarctica (21), has not been reported previously in space.

Rhodotorula mucilaginosa is a ubiquitous environmental (22) and human commensal yeast and opportunistic pathogen (23) that is found in aquatic and built environments, including bathrooms and dishwashers (24, 25). It is the most commonly isolated yeast on the ISS (26–28).

Sample collection and fungal isolation steps were described elsewhere (26). For five flight missions, eight surfaces aboard the ISS were sampled with moistened polyester wipes (Table 1). Upon return to Earth, the wipes were agitated in sterile phosphate-buffered saline, which was concentrated using an InnovaPrep CP150 concentrating pipette, and suitable aliquots were spread onto nutrient media (Table 1). Fungal isolates were restreaked on potato-dextrose agar (PDA), and genomic DNA was extracted using the ZymoBIOMICS MagBead DNA kit according to the manufacturer’s instructions. Whole-genome shotgun sequencing libraries were prepared with an Illumina Nextera DNA Flex library preparation kit (29) and were sequenced on the NovaSeq 6000 paired-end 2 × 150-bp platform with a S4 flow cell. After quality filtering and trimming with FastQC v0.11.7 (30) and fastp v0.20.0 (31), genomes were assembled using SPAdes v3.11.1 (32). Assembly quality was assessed with QUAST v5.0.2 (33). Fastp included screening for 512 adapters; otherwise, default settings were used for all steps.

**TABLE 1:** Sampling locations, genetic loci used for taxonomic analysis, and WGS assembly quality for fungal species isolated from the ISS during the Microbial Tracking-2 mission

Sample name	Fungal species	Loci used for identification^*a*^	WGS accession no.	SRA accession no.	Medium and temperature^*b*^	Flight no.	Location description^*c*^	No. of contigs	Genome size (bp)	*N*_50_ (bp)	Coverage depth (×)	G+C content (%)	No. of filtered reads
F6_8S_P_2A	Aspergillus unguis	*benA*, *CaM*	JAGUQD000000000	SRR14342084	BA, 37°C	F6	Crew quarters	22	25,891,216	2,495,528	84.28	50.30	14,548,376
F6_8S_P_4A	Aspergillus unguis	*benA*, *CaM*	JAGUQC000000000	SRR14342083	BA, 37°C	F6	Crew quarters	19	25,892,532	2,741,542	179.59	50.30	30,999,366
F7_6S_YPD	Aureobasidium pullulans	ITS	JAGUPW000000000	SRR14342072	YPD, 25°C	F7	PPM port 1	105	28,552,932	777,727	77.27	50.35	15,555,668
F7_5S_YPD	Aureobasidium pullulans	ITS	JAGUPX000000000	SRR14342073	YPD, 25°C	F7	Overhead 4	96	28,546,471	780,810	91.03	50.35	18,326,654
F7_2S_YPD	Aureobasidium pullulans	ITS	JAGUPY000000000	SRR14342074	YPD, 25°C	F7	WHC	110	28,545,478	869,212	112.63	50.35	22,675,694
F7_1S_YPD	Aureobasidium pullulans	ITS	JAGUQA000000000	SRR14342076	YPD, 25°C	F7	Cupola	103	28,553,405	847,007	148.55	50.34	29,906,446
F7_2A_YPD	Aureobasidium pullulans	ITS	JAGUPZ000000000	SRR14342075	YPD, 25°C	F7	WHC	102	28,545,383	869,723	90.19	50.35	18,158,278
F7_1A_YPD	Aureobasidium pullulans	ITS	JAGUQB000000000	SRR14342077	YPD, 25°C	F7	Cupola	99	28,555,273	793,969	102.69	50.34	20,674,178
F6_1S_B_1B	Aureobasidium pullulans	ITS	JAGUQJ000000000	SRR14342071	R2A, 25°C	F6	Cupola	162	28,763,896	746,636	72.11	50.32	14,517,038
F6_1S_P_3A	Aureobasidium pullulans	ITS	JAGUQI000000000	SRR14342125	BA, 37°C	F6	Cupola	142	28,780,047	881,182	177.55	50.32	35,744,148
F6_4S_B_1	Aureobasidium pullulans	ITS	JAGUQE000000000	SRR14342118	R2A, 25°C	F6	Dining table	140	28,772,883	884,258	97.41	50.32	19,610,724
F6_3S_1A_F	Aureobasidium pullulans	ITS	JAGUQH000000000	SRR14342124	PDA, 25°C	F6	ARED	156	28,770,985	819,420	133.99	50.32	26,975,786
F6_3S_1B_F	Aureobasidium pullulans	ITS	JAGUQG000000000	SRR14342123	PDA, 25°C	F6	ARED	184	28,720,136	734,664	106.49	50.35	21,439,104
F6_3S_1C_F	Aureobasidium pullulans	ITS	JAGUQF000000000	SRR14342122	PDA, 25°C	F6	ARED	188	28,737,399	748,713	78.37	50.33	15,778,598
F8_5S_2F	Cladosporium cladosporioides	*TEF*	JAGUPV000000000	SRR14342051	PDA, 25°C	F8	Overhead 4	130	34,025,119	1,102,510	120.93	52.59	26,786,080
F8_5S_3F	Cladosporium cladosporioides	*TEF*	JAGUPU000000000	SRR14342048	PDA, 25°C	F8	Overhead 4	132	34,027,712	965,801	122.78	52.59	27,195,854
F8_5S_4F	Cladosporium cladosporioides	*TEF*	JAGUPT000000000	SRR14342047	PDA, 25°C	F8	Overhead 4	234	33,871,387	634,743	111.02	52.67	24,592,420
F4_7S_F1_F	Cladosporium sphaerospermum	*TEF*	JAHARS000000000	SRR14342126	PDA, 25°C	F4	Lab 3 overhead	573	30,616,838	873,859	135.80	53.05	24,348,718
F8_4S_2B	Fusarium annulatum	*TEF*, *RPB2*	JAHAPR000000000	SRR14342059	R2A, 25°C	F8	Dining table	275	45,009,810	1,875,762	42.45^*d*^	48.32	21,486,110
F8_4S_3B	Fusarium annulatum	TEF, *RPB2*	JAHAPP000000000	SRR14342057	R2A, 25°C	F8	Dining table	273	45,012,509	1,556,966	51.28^*d*^	48.32	25,765,430
F8_4S_4P	Fusarium annulatum	*TEF*, *RPB2*	JAHAPN000000000	SRR14342055	BA, 37°C	F8	Dining table	283	45,010,147	1,599,633	40.99^*d*^	48.32	20,595,810
F8_4S_5P	Fusarium annulatum	*TEF*, *RPB2*	JAHAPL000000000	SRR14342053	BA, 37°C	F8	Dining table	309	45,001,499	1,598,178	47.32^*d*^	48.33	23,616,132
F8_4S_1F	Fusarium annulatum	*TEF*, *RPB2*	JAHAPT000000000	SRR14342062	PDA, 25°C	F8	Dining table	341	44,584,936	1,556,345	51.36^*d*^	48.62	25,159,054
F5_8S_1A_F	Fusarium veterinarium	*TEF*	JAHARR000000000	SRR14342093	PDA, 25°C	F5	Crew quarters	861	48,079,010	325,419	58.78^*d*^	47.63	31,171,132
F5_8S_1B_F	Fusarium veterinarium	*TEF*	JAHARQ000000000	SRR14342082	PDA, 25°C	F5	Crew quarters	925	47,312,867	276,643	44.14^*d*^	48.09	23,238,836
F4_1A_F1_F	Penicillium citrinum	*benA*, *CaM*	JAHART000000000	SRR14342127	PDA, 25°C	F4	Cupola	88	31,021,730	1,108,866	127.62	46.39	26,826,262
F5_1S_1A_F	Penicillium corylophilum	*benA*, *CaM*	JAGUQL000000000	SRR14342115	PDA, 25°C	F5	Cupola	53	28,229,796	1,725,911	104.56^*d*^	50.20	32,206,152
F5_1S_1B_F	Penicillium corylophilum	*benA*, *CaM*	JAGUQK000000000	SRR14342104	PDA, 25°C	F5	Cupola	53	28,231,333	1,604,566	80.64^*d*^	50.20	24,805,488
F6_4S_1A_F	Penicillium palitans	*benA*, *CaM*	JAHARM000000000	SRR14342121	PDA, 25°C	F6	Dining table	798	36,471,070	281,544	57.4^*d*^	47.70	23,233,174
F6_4S_1B_F	Penicillium palitans	*benA*, *CaM*	JAHARL000000000	SRR14342120	PDA, 25°C	F6	Dining table	806	36,468,364	273,565	58.94^*d*^	47.70	24,107,708
F6_4S_1C_F	Penicillium palitans	*benA*, *CaM*	JAHARK000000000	SRR14342119	PDA, 25°C	F6	Dining table	802	36,477,181	268,530	61.99^*d*^	47.70	25,189,046
F6_6S_1_F	Penicillium palitans	*benA*, *CaM*	JAHARA000000000	SRR14342107	PDA, 25°C	F6	PPM port 1	808	36,467,611	268,531	64.47^*d*^	47.70	26,242,930
F6_7S_1A_F	Penicillium palitans	*benA*, *CaM*	JAHAQR000000000	SRR14342097	PDA, 25°C	F6	Lab 3 overhead	820	36,439,435	268,531	57.88^*d*^	47.70	23,443,712
F6_7S_1C_F	Penicillium palitans	*benA*, *CaM*	JAHAQQ000000000	SRR14342096	PDA, 25°C	F6	Lab 3 overhead	794	36,471,119	303,786	69.09^*d*^	47.70	28,047,394
F6_8S_1A_F	Penicillium palitans	*benA*, *CaM*	JAHAQI000000000	SRR14342087	PDA, 25°C	F6	Crew quarters	828	36,453,009	268,529	57.59^*d*^	47.70	23,229,736
F6_8S_1C_F	Penicillium palitans	*benA*, *CaM*	JAHAQH000000000	SRR14342086	PDA, 25°C	F6	Crew quarters	850	36,588,697	255,978	57.23^*d*^	47.70	22,858,702
F8_6S-1F	Penicillium palitans	*benA*, *CaM*	JAHAOZ000000000	SRR14342036	PDA, 25°C	F8	PPM port 1	987	36,400,448	262,197	53.41^*d*^	47.80	21,366,974
F8_6S_2F	Penicillium palitans	*benA*, *CaM*	JAHAPD000000000	SRR14342041	PDA, 25°C	F8	PPM port 1	943	36,503,579	273,760	70.93^*d*^	47.76	28,334,002
F8_6S-3F	Penicillium palitans	*benA*, *CaM*	JAHAOY000000000	SRR14342035	PDA, 25°C	F8	PPM port 1	799	36,607,893	290,808	60.5^*d*^	47.69	24,779,924
F8_6S-4F	Penicillium palitans	*benA*, *CaM*	JAHAOX000000000	SRR14342034	PDA, 25°C	F8	PPM port 1	827	36,605,405	290,729	60.84^*d*^	47.69	24,966,852
F8_6S_5F	Penicillium palitans	*benA*, *CaM*	JAHAPC000000000	SRR14342040	PDA, 25°C	F8	PPM port 1	808	36,607,101	281,504	73.76^*d*^	47.70	30,172,808
F8_6S_6F	Penicillium rubens	*benA*, *CaM*	JAHAPB000000000	SRR14342039	PDA, 25°C	F8	PPM port 1	182	30,459,701	858,760	118.82	49.00	26,445,782
F8_6S_7F	Penicillium rubens	*benA*, *CaM*	JAHAPA000000000	SRR14342037	PDA, 25°C	F8	PPM port 1	469	31,558,626	716,780	112.63	48.97	25,069,320
F6_4S_B_2B	Rhodotorula mucilaginosa	ITS	JAHARI000000000	SRR14342116	R2A, 25°C	F6	Dining table	199	20,171,565	432,962	132.05	60.55	17,800,488
F6_8S_B_1B	Rhodotorula mucilaginosa	ITS	JAHAQG000000000	SRR14342085	R2A, 25°C	F6	Crew quarters	209	20,172,702	367,952	215.04	60.55	28,987,090
F6_8S_P_5A	Rhodotorula mucilaginosa	ITS	JAHAQF000000000	SRR14342081	BA, 37°C	F6	Crew quarters	199	20,077,383	389,367	212.72	59.99	28,674,716
F6_8S_P_5B	Rhodotorula mucilaginosa	ITS	JAHAQE000000000	SRR14342080	BA, 37°C	F6	Crew quarters	194	20,074,587	329,841	244.78	59.99	32,995,640
F6_8S_P_6A	Rhodotorula mucilaginosa	ITS	JAHAQD000000000	SRR14342079	BA, 37°C	F6	Crew quarters	201	20,175,089	436,573	227.88	60.55	30,717,298
F6_8S_P_6B	Rhodotorula mucilaginosa	ITS	JAHAQC000000000	SRR14342078	BA, 37°C	F6	Crew quarters	213	20,169,473	376,635	52.49	60.55	7,075,538
F8_5S_4P	Rhodotorula mucilaginosa	ITS	JAHAPI000000000	SRR14342046	BA, 37°C	F8	Overhead 4	202	20,109,607	317,098	232.29	60.53	31,313,072
F8_5S_5P	Rhodotorula mucilaginosa	ITS	JAHAPH000000000	SRR14342045	BA, 37°C	F8	Overhead 4	195	20,116,897	319,540	215.19	60.53	29,006,980
F8_5S_6P	Rhodotorula mucilaginosa	ITS	JAHAPG000000000	SRR14342044	BA, 37°C	F8	Overhead 4	190	20,123,419	354,782	251.44	60.53	33,893,490
F6_1S_P_1A	Rhodotorula mucilaginosa	ITS	JAHARP000000000	SRR14342060	BA, 37°C	F6	Cupola	179	20,117,026	392,515	180.21	60.53	24,292,752
F6_1S_P_1B	Rhodotorula mucilaginosa	ITS	JAHARO000000000	SRR14342049	BA, 37°C	F6	Cupola	182	20,114,730	392,620	225.92	60.53	30,453,336
F6_1S_P_1C	Rhodotorula mucilaginosa	ITS	JAHARN000000000	SRR14342038	BA, 37°C	F6	Cupola	200	20,115,701	322,180	218.74	60.53	29,485,618
F6_4S_B_2A	Rhodotorula mucilaginosa	ITS	JAHARJ000000000	SRR14342117	R2A, 25°C	F6	Dining table	172	19,998,495	334,411	207.28	60.55	27,941,724
F6_4S_B_2C	Rhodotorula mucilaginosa	ITS	JAHARH000000000	SRR14342114	R2A, 25°C	F6	Dining table	200	20,107,055	319,541	200.96	60.53	27,089,466
F6_4S_P_3B	Rhodotorula mucilaginosa	ITS	JAHARG000000000	SRR14342113	BA, 37°C	F6	Dining table	184	20,108,582	331,731	105.50	60.53	14,221,722
F6_4S_P_3C	Rhodotorula mucilaginosa	ITS	JAHARF000000000	SRR14342112	BA, 37°C	F6	Dining table	196	20,111,077	317,326	150.66	60.53	20,308,150
F6_4S_P_4A	Rhodotorula mucilaginosa	ITS	JAHARE000000000	SRR14342111	BA, 37°C	F6	Dining table	175	20,126,113	410,825	121.23	60.52	16,341,478
F6_4S_P_4B	Rhodotorula mucilaginosa	ITS	JAHARD000000000	SRR14342110	BA, 37°C	F6	Dining table	206	20,115,791	297,628	230.74	60.53	31,103,222
F6_4S_P_5A	Rhodotorula mucilaginosa	ITS	JAHARC000000000	SRR14342109	BA, 37°C	F6	Dining table	195	20,113,572	334,410	220.75	60.53	29,757,146
F6_4S_P_5B	Rhodotorula mucilaginosa	ITS	JAHARB000000000	SRR14342108	BA, 37°C	F6	Dining table	210	20,109,987	323,162	141.10	60.53	19,019,728
F6_6S_B_1A	Rhodotorula mucilaginosa	ITS	JAHAQZ000000000	SRR14342106	R2A, 25°C	F6	PPM port 1	202	20,109,799	329,302	205.40	60.53	27,687,960
F6_6S_B_1B	Rhodotorula mucilaginosa	ITS	JAHAQY000000000	SRR14342105	R2A, 25°C	F6	PPM port 1	199	20,106,114	331,797	130.16	60.53	17,545,556
F6_6S_B_1C	Rhodotorula mucilaginosa	ITS	JAHAQX000000000	SRR14342103	R2A, 25°C	F6	PPM port 1	199	20,115,093	335,579	133.73	60.53	18,026,650
F6_6S_P_1A	Rhodotorula mucilaginosa	ITS	JAHAQW000000000	SRR14342102	BA, 37°C	F6	PPM port 1	202	20,108,679	319,902	114.06	60.53	15,375,556
F6_6S_P_1B	Rhodotorula mucilaginosa	ITS	JAHAQV000000000	SRR14342101	BA, 37°C	F6	PPM port 1	197	20,116,515	323,169	233.47	60.53	31,471,482
F6_6S_P_1C	Rhodotorula mucilaginosa	ITS	JAHAQU000000000	SRR14342100	BA, 37°C	F6	PPM port 1	190	20,114,955	329,677	145.50	60.53	19,613,002
F6_6S_P_2A	Rhodotorula mucilaginosa	ITS	JAHAQT000000000	SRR14342099	BA, 37°C	F6	PPM port 1	191	20,108,060	323,034	240.93	60.53	32,476,452
F6_6S_P_2B	Rhodotorula mucilaginosa	ITS	JAHAQS000000000	SRR14342098	BA, 37°C	F6	PPM port 1	194	20,117,485	323,058	209.85	60.52	28,287,446
F6_7S_B_2A	Rhodotorula mucilaginosa	ITS	JAHAQP000000000	SRR14342095	R2A, 25°C	F6	Lab 3 overhead	183	20,055,075	293,584	240.80	60.55	32,459,440
F6_7S_B_2B	Rhodotorula mucilaginosa	ITS	JAHAQO000000000	SRR14342094	R2A, 25°C	F6	Lab 3 overhead	192	20,049,052	293,667	138.13	60.55	18,619,460
F6_7S_B_2C	Rhodotorula mucilaginosa	ITS	JAHAQN000000000	SRR14342092	R2A, 25°C	F6	Lab 3 overhead	196	20,050,303	292,236	107.06	60.55	14,431,658
F6_7S_P_6B	Rhodotorula mucilaginosa	ITS	JAHAQM000000000	SRR14342091	BA, 37°C	F6	Lab 3 overhead	164	20,065,557	369,724	234.38	60.55	31,594,198
F6_7S_P_7A	Rhodotorula mucilaginosa	ITS	JAHAQL000000000	SRR14342090	BA, 37°C	F6	Lab 3 overhead	199	20,052,658	311,987	201.97	60.55	27,225,956
F6_7S_P_7B	Rhodotorula mucilaginosa	ITS	JAHAQK000000000	SRR14342089	BA, 37°C	F6	Lab 3 overhead	198	20,056,236	284,145	169.60	60.55	22,862,414
F6_7S_P_7C	Rhodotorula mucilaginosa	ITS	JAHAQJ000000000	SRR14342088	BA, 37°C	F6	Lab 3 overhead	190	20,058,305	320,873	233.38	60.55	31,459,534
F8_1S_2B	Rhodotorula mucilaginosa	ITS	JAHAQB000000000	SRR14342070	R2A, 25°C	F8	Cupola	172	19,998,942	329,308	180.71	60.55	24,358,996
F8_1S_3B	Rhodotorula mucilaginosa	ITS	JAHAQA000000000	SRR14342069	R2A, 25°C	F8	Cupola	197	20,118,130	319,542	217.40	60.53	29,305,254
F8_3S_1B	Rhodotorula mucilaginosa	ITS	JAHAPZ000000000	SRR14342068	R2A, 25°C	F8	ARED	197	20,118,712	325,275	190.77	60.53	25,716,020
F8_3S_2B	Rhodotorula mucilaginosa	ITS	JAHAPX000000000	SRR14342066	R2A, 25°C	F8	ARED	200	20,109,774	323,187	181.13	60.53	24,415,874
F8_3S_3B	Rhodotorula mucilaginosa	ITS	JAHAPV000000000	SRR14342064	R2A, 25°C	F8	ARED	189	20,104,868	317,674	194.41	60.53	26,206,654
F8_4S_4B	Rhodotorula mucilaginosa	ITS	JAHAPO000000000	SRR14342056	R2A, 25°C	F8	Dining table	172	19,998,636	338,336	224.66	60.54	30,283,760
F8_4S_5B	Rhodotorula mucilaginosa	ITS	JAHAPM000000000	SRR14342054	R2A, 25°C	F8	Dining table	172	19,999,954	323,188	197.65	60.55	26,642,384
F8_5S_2B	Rhodotorula mucilaginosa	ITS	JAHAPK000000000	SRR14342052	R2A, 25°C	F8	Overhead 4	189	20,113,164	335,288	219.00	60.53	29,520,936
F8_5S_3B	Rhodotorula mucilaginosa	ITS	JAHAPJ000000000	SRR14342050	R2A, 25°C	F8	Overhead 4	199	20,113,962	333,776	192.52	60.53	25,952,066
F8_6S_1B	Rhodotorula mucilaginosa	ITS	JAHAPF000000000	SRR14342043	R2A, 25°C	F8	PPM port 1	169	19,995,004	329,309	187.59	60.54	25,287,420
F8_6S_2B	Rhodotorula mucilaginosa	ITS	JAHAPE000000000	SRR14342042	R2A, 25°C	F8	PPM port 1	179	19,994,438	397,651	188.04	60.55	25,347,104
F8_3S_1P	Rhodotorula mucilaginosa	ITS	JAHAPY000000000	SRR14342067	BA, 37°C	F8	ARED	183	19,994,621	322,715	192.67	60.55	25,972,138
F8_3S_2P	Rhodotorula mucilaginosa	ITS	JAHAPW000000000	SRR14342065	BA, 37°C	F8	ARED	167	19,991,553	415,431	121.35	60.54	16,358,020
F8_3S_3P	Rhodotorula mucilaginosa	ITS	JAHAPU000000000	SRR14342063	BA, 37°C	F8	ARED	193	20,115,201	319,541	196.12	60.53	26,437,390
F8_4S_1P	Rhodotorula mucilaginosa	ITS	JAHAPS000000000	SRR14342061	BA, 37°C	F8	Dining table	171	19,999,629	322,714	190.79	60.55	25,717,964
F8_4S_2P	Rhodotorula mucilaginosa	ITS	JAHAPQ000000000	SRR14342058	BA, 37°C	F8	Dining table	173	19,998,413	415,991	257.98	60.54	34,775,980

a *benA*, β-tubulin; *CaM*, calmodulin; *RPB2*, DNA-directed RNA polymerase II subunit; *TEF*, translation elongation factor 1.

b BA, blood agar; R2A, Reasoner’s 2A agar; YPD, yeast extract-peptone-dextrose.

c ARED, advanced resistive exercise device; WHC, waste and hygiene compartment; PMM, permanent multipurpose module.

d Reference genome was not available; average sequencing depth was calculated from *k*-mer coverage.

Genus-level identification was made via BLAST searches against the UNITE nuclear ribosomal internal transcribed spacer (ITS) database (34). Species identification was performed using specific loci suitable for species recognition (Table 1) (35). Homology searches were performed with extracted sequences against the NCBI nucleotide database and in-house Westerdijk Fungal Biodiversity Institute databases containing reference sequences; in case of doubt, identification was confirmed by constructing phylograms.

### Data availability.

The whole-genome sequences (WGSs) and raw data have been deposited in GenBank under BioProject accession number PRJNA723004. This project has also been deposited in the NASA GeneLab system (36) under project number GLDS-400. The version described in this paper is the first version.
